# Relationship among vaginal palpation, vaginal squeeze pressure, electromyographic and ultrasonographic variables of female pelvic floor muscles

**DOI:** 10.1590/bjpt-rbf.2014.0038

**Published:** 2014

**Authors:** Vanessa S. Pereira, Humberto S. Hirakawa, Ana B. Oliveira, Patricia Driusso

**Affiliations:** 1 Departamento de Fisioterapia, Universidade Federal de São Carlos (UFSCar), São Carlos, SP, Brasil; 2 Departamento de Medicina, UFSCar, São Carlos, SP, Brasil

**Keywords:** pelvic floor, electromyography, physical therapy

## Abstract

**Background:**

The proper evaluation of the pelvic floor muscles (PFM) is essential for choosing the correct treatment. Currently, there is no gold standard for the assessment of female PFM function.

**Objective:**

To determine the correlation between vaginal palpation, vaginal squeeze pressure, and electromyographic and ultrasonographic variables of the female PFM.

**Method:**

This cross-sectional study evaluated 80 women between 18 and 35 years of age who were nulliparous and had no pelvic floor dysfunction. PFM function was assessed based on digital palpation, vaginal squeeze pressure, electromyographic activity, bilateral diameter of the bulbocavernosus muscles and the amount of bladder neck movement during voluntary PFM contraction using transperineal bi-dimensional ultrasound. The Pearson correlation was used for statistical analysis (p<0.05).

**Results:**

There was a strong positive correlation between PFM function and PFM contraction pressure (0.90). In addition, there was a moderate positive correlation between these two variables and PFM electromyographic activity (0.59 and 0.63, respectively) and movement of the bladder neck in relation to the pubic symphysis (0.51 and 0.60, respectively).

**Conclusions:**

This study showed that there was a correlation between vaginal palpation, vaginal squeeze pressure, and electromyographic and ultrasonographic variables of the PFM in nulliparous women. The strong correlation between digital palpation and PFM contraction pressure indicated that perineometry could easily be replaced by PFM digital palpation in the absence of equipment.

## Introduction

The pelvic floor muscles (PFM) form the base of the pelvis and abdominal cavity^1^. These muscles are intimately involved in the function of the lower urinary and anorectal tract, in sexual function^2^, and in the stabilization of the spine and pelvis by indirectly aiding lumbopelvic stabilization^3^.

Pelvic floor dysfunction affects approximately 50% of women over 50 years of age and may present as urinary or fecal incontinence, chronic constipation, pelvic pain and pelvic organ prolapse^4^. These dysfunctions have a significant impact on women's quality of life, and the costs of care are a concern to governmental institutions^5^^,^ 6. A recent study projected that the need to care for women with pelvic floor dysfunctions will increase by 35% between 2010 and 2030 in the United States if population growth continues^7^.

Given the high prevalence and costs involved in the care of women with pelvic floor dysfunction, it is essential to develop effective and low-cost treatments^8^. Positive results from physical therapy in women with pelvic floor dysfunctions have been reported^9^^-^ 11. Conducting proper evaluations is essential for the development of effective treatments. Studies have indicated that treatment failure in women with pelvic floor dysfunction is more frequently caused by an incorrect evaluation than by inadequate therapy^12^.

Thus, evaluation of the PFM is essential for the development and success of appropriate treatments. Currently, there is no evaluation tool that is considered the gold standard, which makes the comparison of results difficult and imprecise^13^. The International Continence Society recommends that functional evaluation of the pelvic floor should be performed through visual inspection, digital palpation, perineometry or electromyography^14^. These are important evaluation methods but do not provide direct information about the anatomy of the region^4^. Thus, evaluation methods using imaging, such as ultrasound and magnetic resonance, have also been proposed^4^^,^ 15.

Little is known about the correlation between imaging methods and other forms of PFM evaluation. Therefore, the objective of this study was to verify the correlation between digital palpation, contraction pressure, EMG activity and sonographic variables of the PFM.

## Method

This study was conducted at the Laboratory for Research on Women's Health (Laboratório de Pesquisa em Saúde da Mulher), at Universidade Federal de São Carlos (UFScar) in São Carlos, SP, Brazil between January 2012 and July 2013. For this cross-sectional study, women between 18 and 35 years of age who were nulliparous with no reported PFM dysfunction were recruited from the community. Exclusion criteria consisted of an inability to voluntarily contract the PFM, body mass index greater than 25 kg/cm^2^, previous urogynecological surgery, previous PFM training or the presence of any cognitive impairment or neurological condition that could influence muscle activation. To ensure that all volunteers were able to perform a voluntary contraction of the muscles of interest, a functional evaluation of PFM was performed using digital palpation. Muscle function was classified according to the Modified Oxford Scale^16^, and these women classified with zero function (absence of muscle response) were excluded. All participants signed a free and informed consent, and the study was approved by the Ethics Committee on Human Research of the Centro Universitário Central Paulista (UNICEP) in São Carlos, SP, Brazil (protocol number 020/2011).

A sample size calculation was performed using the *GPower* software version 3.1 with perineometry correlation data on the displacement of the bladder neck relative to the pubic symphysis during PFM contraction (r=0.43) from Thompson et al.^17^. According to this calculation, a sample of 74 women would be required to achieve a statistical power of 95% at a significance level of 3%.

## Procedures

All subjects underwent initial anamnesis and physical examination that was performed by a single physical therapist with previous experience. Before starting the study, evaluation reproducibility was verified by the responsible physical therapist. A total of 15 women were evaluated on two occasions one week apart to determine the intraclass correlation coefficient (ICC) of all variables.

### Digital palpation and contraction pressure of the pelvic floor muscles

PFM evaluation was initially performed by digital palpation as proposed by Laycock and Jerwood^16^. For this purpose, the volunteers were positioned supine with flexed hips and knees. The therapist introduced the index finger approximately 4 cm inside the vagina and asked the volunteer to perform a maximum PFM contraction, instructing them to make an "inwards and upwards" movement with the greatest possible force. Muscle function was classified according to the Modified Oxford Scale, ranging from zero (no muscle contraction) to five (strong contraction with suction of the evaluator's finger) (ICC: 0.99).

PFM contraction pressure was evaluated using *Peritron* equipment (*Cardio Design Pty Ltd,* Oakleigh, Victoria, Australia) equipped with a vaginal probe. For this measurement, volunteers remained in the aforementioned position and the vaginal sensor was inserted approximately 3.5 cm into the vaginal cavity. Next, the device was calibrated. Volunteers were instructed verbally to perform three contractions of the PFM with the greatest possible force, each lasting for three seconds with a one-minute rest interval. The achievement of a correct contraction was visually verified by the physical therapist. All volunteers were instructed to avoid use of the abdominal, gluteal and hip adductor muscles^18^. The average of three valid contractions was used for data analysis (ICC: 0.97).

### Electromyographic evaluation

A *MyoTrac Infiniti (Thought* Technology Ltd, Canada) electromyograph was used to collect electromyographic data (acquisition frequency: 1000 Hz; gain accuracy: 0.5%; input impedance: 10 GW; analog bandpass filter 20-500 Hz; Butterworth *anti-aliasing* Filter, 4^th^ order 500 Hz; CMRR> 130 dB; gain: 500). The volunteers were asked to remain in the supine position with flexed hips and knees. An intravaginal sensor (AS 9572, *Thought Technology* Ltd, Canada) comprised of two stainless steel lateral electrodes (length 3.5 cm and width 1.0 cm) was used to capture the data. The sensor was inserted 3.5 cm into the vaginal cavity, and the plates remained side-to-side. The reference electrode (*Medi-Trace(tm)*, Kendall, Mansfield, MA, USA) was positioned on the right anterior superior iliac crest of the volunteer.

PFM electromyographic activity was collected during the performance of abdominal contractions to normalize the EMG data^19^. To perform abdominal contractions, the volunteers were instructed to remain with the hips and knees flexed at 45 degrees and to make a slight attempt to sit up^20^ by removing the head and upper portion of the shoulder blades and to maintain this position for five seconds before returning to the original position. A familiarization maneuver and three valid maneuvers were performed, each lasting for five seconds with a one-minute rest interval. No instruction regarding PFM contraction was given during the task.

After ten minutes, the volunteers were instructed to perform a maximal voluntary PFM contraction with the same instruction to move "inward and upward" with the greatest possible force and to hold the contraction until they experienced a maximum feeling of fatigue. The volunteer was instructed to report feelings of fatigue by the word "yes" and then to remain relaxed until the end of collection time. If the volunteers completed a minute of contraction without reporting fatigue, the examiner directed them to stop the contraction.

Processing of the EMG data was accomplished using routine programs with *Matlab* (v. R2008a, *MathWorks*, Natick, MA). Initially a *Butterworth* digital filter was applied with a bandpass of 20-450 Hz and 4th order zero phase lag. The data were then transformed into *root mean square* (RMS) values using windowing. The windows were programmed for a duration of 40 ms and 50% overlap. For the abdominal contraction, the mean RMS value was computed and considered as the mean voluntary electrical activity for each of the three contractions. The mean value of the three contractions was then calculated. For voluntary contraction of the PFM to fatigue, the initial five seconds of contraction were used, and the maximum value of that period was calculated.

To normalize the data, the maximum RMS value of the initial five seconds of voluntary contraction until fatigue was divided by the mean value of the abdominal contraction maneuver activity and then expressed as a percentage of electrical activity (ICC: 0.95).

### Sonographic evaluation

After an interval of five to seven days, sonographic evaluation of the pelvic floor was performed. The evaluation was conducted using a transperineal technique with *Venue 40* two-dimensional equipment (*GE Healthcare*, Waukesha, WI, USA) coupled to a convex transducer (2 to 5.5 MHz) by a physician sonographer with previous experience. The volunteers were instructed to empty their bladder one hour before the exam and then to drink 500 ml of water and refrain from further urinating until the examination^17^. All examinations were performed with bladder contents of 50-250 ml as measured by ultrasound.

The volunteer was positioned supine with flexed hips and knees. A bilateral measurement of the greatest side-to-side diameter of the bulbocavernosus muscle was determined in centimeters^21^. Three measurements were taken at rest, and the mean of the measurements was used for analysis (ICC right diameter: 0.94; ICC left diameter: 0.90). The distance between the pubic symphysis and the bladder neck was then measured. Three measurements at rest and three measurements during performance of maximal voluntary PFM contractions were made^17^, with one-minute rest intervals between contractions. The mean measurement was calculated, and the difference between the distance during contraction and at rest was used for analysis (ICC: 0.81).

### Statistical analysis

Statistical analysis was performed using the *Statistical Package for Social Sciences* software (SPSS V17, Chicago, IL). Data normality was tested using the Shapiro-Wilk test. The intraclass correlation coefficient (ICC(2, k)) was calculated to analyze the reproducibility of the evaluation methods. ICC values greater than 0.75 were considered excellent^22^.

The Pearson correlation test was used to verify the correlation between variables. A significance level of p<0.05 was used. Correlation values were interpreted according to the following guidelines: 0.00 to 0.19 = none to slight; 0.20 to 0.39 = slight; 0.40 to 0.69 = moderate; = 0.70 to 0.89 = high and 0.90 to 1.00 = very high^23^. Data are expressed as the mean and standard deviation (SD).

## Results

A total of 82 women were selected for this study. Among those selected, two were excluded due to their inability to perform voluntary PFM contractions. A total of 80 volunteers completed the study with a mean age of 25.7 (SD: 4.5) years and mean body mass index of 20.9 (SD: 1.8) kg/m^2^.

Digital palpitation evaluation revealed a mean of 2.71 for PFM function (SD: 0.90). The contraction pressure and RMS normalized by mean abdominal contraction were 51.14 (SD: 24.87) cmH_2_O and 520.0 (SD: 324.0)%, respectively. Sonography revealed means of 1.25 (SD: 0.22) cm and 1.23 (SD: 0.22) cm for the diameters of the right and left bulbocavernosus muscles, respectively. The volunteers showed a mean value of 0.27 (SD: 0.22) cm for the difference in distance from the bladder neck to the pubic symphysis during maximal voluntary contraction and at rest.

A correlation analysis performed between variables showed a strong positive correlation between PFM function and contraction pressure. There was a moderate positive correlation between these two variables and RMS normalized by abdominal contraction, as well as the displacement of the bladder neck relative to the pubic symphysis. There was also a slight negative correlation between electromyographic variables and the diameters of the left and right bulbocavernosus muscles (Table 1).

**Table 1:** Pearson Product-Moment Correlation Coefficients.

	Vaginal squeeze pressure (cmH2O)	RMS normalized	Right bulbocavernosus diameter (cm)	Right bulbocavernosus diameter (cm)	Bladder neck movement (cm)
Pelvic floor muscles function	0.90*	0.59*	–0.15	–0.13	0.51*
Vaginal squeeze pressure (cmH_2_O)		0.63*	–0.10	–0.08	0.60*
RMS normalized			–0.27*	–0.22*	0.32
Right muscular diameter (cm)				0.92*	0.12
Left muscular diameter (cm)					0.13

* p<0.05.

## Discussion

Among the methods of imaging evaluation, ultrasonography has gained prominence because it is a simple, safe and low-cost technique that does not use radiation^15^. In the present study, we found that the extent of bladder neck displacement relative to the pubic symphysis during contraction of the PFM positively correlated with muscle function as evaluated by digital palpation and PFM contraction pressure in young nulliparous women.

Thompson et al.^17^ and Dietz et al.^24^ found a moderate positive correlation between the same variables evaluated in the present study. The normal function of the PFM is defined as the ability to perform a normal or strong voluntary contraction, the presence of an involuntary contraction that results in the circular closure of the vagina, urethra and anus and a cranio-ventral movement of the perineum with a rise of the pelvic organs^25^. As observed in the present study, a muscle with greater contraction capacity should promote greater cranial displacement.

Despite the ease of use and low cost of PFM evaluation using digital palpation and perineometry^26^, these techniques are not appropriate for all populations. Some women experience intolerance to vaginal introduction, or introduction is inappropriate, as in the case of children^1^. The results found in this and previous studies demonstrated that transperineal ultrasonography may be an option for PFM evaluation and for education on the correct contraction of these muscles.

Contrary to expectations, only a slight negative correlation was found between the bilateral diameter of the bulbocavernosus muscle and the RMS for PFM contraction normalized by an abdominal contraction. No significant correlation was found between muscle diameter and other variables. Studies have shown that for some skeletal muscles, there is a direct relationship between the cross-sectional muscle area and generation of force^27^. However, this information is unknown for the PFM. Mørkved et al.^28^ evaluated nulliparous women in the second trimester of pregnancy by means of a three-dimensional transperineal ultrasound and found a strong correlation between the thickness of the urogenital diaphragm and PFM contraction pressure measured by perineometry. Braekken et al.^29^ found that clinical PFM variables, such as contraction pressure, explained only 26% of muscle thickness in women with pelvic organ prolapse. According to these authors, genetically determined architecture, involuntary function, level of muscle training and the presence of muscular injuries may relate to a higher percentage of muscle thickness.

One must also consider that PFM have peculiar characteristics because they are involved in organ support and lumbopelvic stability^30^. The PFM contribute to the activities of the spine and pelvis through co-contraction with transverse abdominus, internal oblique, external oblique and rectus abdominis muscles. The PFM are therefore recruited for different tasks related to posture as well as breathing and various activities of daily living^3^. The morphometry and histochemistry of the PFM in humans have demonstrated a predominance of type I fibers with tonic function^31^. However, the PFM have a lower average diameter of type I fibers compared with other non-pelvic tonic muscles^2^. It is therefore possible that the correlation between muscle area and the ability to generate force that has been shown in other muscles^27^ does not apply to the PFM.

In the present study, a moderate positive correlation was observed between the electromyographic variables and the PFM contraction pressure and function variables. Surface PFM electromyography has been widely used for the evaluation of the neuromuscular function of these muscles and to gain a better understanding of muscle function during different activities^32^. Studies with other skeletal muscles have indicated that there is a relationship between the level of muscle strength and electromyographic activity^33^. This relationship also appears to apply to PFM. Botelho et al.^13^ observed a strong positive correlation between non-normalized EMG activity in microvolts and digital palpation graded by the Modified Oxford Scale.

Digital palpation is widely used in clinical practice because it is a simple evaluation technique that does not require equipment. However, this evaluation technique is highly dependent on the physical therapist's experience^26^. The strong correlation found in the present study between the values of digital palpation and PFM contraction pressure has been well described in previous studies^34^^,^ 35. The results therefore indicate that in the absence of equipment, perineometry can be easily replaced by digital palpation of the PFM when performed by an experienced physical therapist.

As for other evaluation methods, the results of the present study indicate that physical therapists should exercise caution when choosing evaluation methods because the moderate to weak correlation found between the different methods indicates that multiple techniques may be needed for a proper PFM evaluation. Moreover, measuring the displacement of the bladder neck with respect to the pubic symphysis using ultrasonography is an option for evaluating muscle function in women when vaginal introduction is not recommended.

This study was limited by the use of two-dimensional ultrasonography. In Brazil, two-dimensional devices are not available at all health centers. Equipment with 3D and 4D technologies is complex and expensive and restricted to large diagnostic centers. However, new technologies allow for a more accurate evaluation of muscle thickness. Further studies should be performed to verify the clinical applicability of the new 3D and 4D technologies.

The results of this study are limited to a population of young, nulliparous and eutrophic women. Additional studies should be conducted that compare PFM evaluations from different techniques in other populations, such as incontinent, elderly or pregnant women.

## Conclusions

The present study showed that there was a correlation between sonographic variables and muscle function, and between contraction pressure and electromyographic activity of the PFM in young nulliparous women. In addition, there was a strong correlation between muscle function as determined by digital palpation and contraction pressure, indicating that, digital palpation could replace the use of pressure measurement equipment in clinical practice.
